# *Mycobacterium bovis* Isolates from Tuberculous Lesions in Chadian Zebu Carcasses

**DOI:** 10.3201/eid1205.050691

**Published:** 2006-05

**Authors:** Colette Diguimbaye-Djaibé, Markus Hilty, Richard Ngandolo, Hassane H. Mahamat, Gaby E. Pfyffer, Franca Baggi, Glyn Hewinson, Marcel Tanner, Jakob Zinsstag, Esther Schelling

**Affiliations:** * Laboratoire de Recherches Vétérinaires et Zootechniques de Farcha, N'Djaména, Chad;; † Swiss Tropical Institute, Basel, Switzerland; ‡Kantonsspital Luzern, Luzern, Switzerland;; § National Centre for Mycobacteria, Zurich, Switzerland;; ¶ Veterinary Laboratories Agency, Weybridge, United Kingdom

**Keywords:** Mycobacterium bovis, proportion, Arab breed, Mbororo breed, slaughterhouse, molecular typing, Chad, zebu, dispatch

## Abstract

This slaughterhouse study in Chad shows higher proportions of *Mycobacterium bovis* isolates among Mbororo than Arabe zebu cattle. Spoligotyping shows a homogenetic population structure for *M. bovis* and lack of spacer 30, as were found in neighboring Cameroon and Nigeria. This finding suggests transborder and ongoing transmission between cattle.

In Chad, prevalences of tuberculin-positive cattle are 0.8% (95% confidence interval [CI] 0.2%–1.4%) in the east (Ouaddaï region) (*1*) and 16.9% (95% CI 10.4%–23.5%) in the west (Chari-Baguirmi and Kanem regions) (*2*). The latter comparative intradermal tuberculin study was conducted with 34 additional transhumant herds; a prevalence of 11.5% (CI 6.9%–18.5%) was found when herds were considered as random effect in the model. More tuberculin reactors were found among Mbororo than Arab zebus (p = 0.02). In the slaughterhouse of Farcha in N'Djaména, 90% of slaughtered cattle are of the Arab zebu breed, 7% Mbororo zebu, and 3% Kouri (*3*). Previous slaughterhouse studies showed that bovine tuberculosis (TB) is an important cause of condemnation (i.e., if a carcass is fully condemned, the whole carcass is destroyed [≈9% of all inspected cattle carcasses]) (*4*). A retrospective study on causes of condemnation after meat inspection showed that most carcasses with tuberculous lesions were detected from July to November and that more Mbororo cattle than other breeds had TB-like lesions (42/60 vs. 132/1,539) (*5*). The diagnosis of suspected bovine TB was based on sighting of typical macroscopic lesions of the organs during meat inspection.

In Chad, until this study was undertaken, bovine TB was not confirmed by isolation or molecular characterization of the causative agent, *Mycobacterium bovis*. This organism is recognized as a zoonotic pathogen that infects many persons, particularly in the developing world. The highest prevalence of coinfection with bovine TB and HIV/AIDS is also in the developing world (*6*). Our study was aimed at isolating the first *M. bovis* isolates from specimens of Mbororo and Arab cattle in the newly setup mycobacteriology unit of the veterinary laboratory of Fracha, at characterizing the isolates with molecular methods, and at comparing the isolates with those from Cameroon (*7*).

## The Study

From July 1 to August 31, 2002, a total of 727 of 10,000 cattle carcasses at the slaughterhouse of Farcha were condemned because of TB-like lesions on meat inspection. The overall prevalence of suspect lesions was 7.3%. A significantly higher (p = 0.04) proportion of lesions was found among Mbororo (8.2%; 212/2,596) than Arab (7%; 515/7,397) cattle (*8*). Lesions were mainly found in the lymph nodes and lungs (Table).

**Table:** Specimens collected at the main slaughterhouse of N'Djaména, Chad, and specifications of the condemned carcasses

Organ/tissue	n	Condemnation		Breed		Sex	
		Entire	Partial	Arab	Mbororo	Male	Female
Lymph nodes	116	17	99	67	49	8	108
Lungs	75	13	62	51	24	1	74
Lungs and lymph nodes	2	0	2	2	0	0	2
Liver	5	0	5	4	1	0	5
Miliary tuberculosis	1	0	1	0	1	0	1
Total	199	30	169	124	75	9	190

Specimens from 201 affected organs (lymph nodes, lungs, and liver) of 199 randomly selected carcasses were collected for further processing along with the following information: breed, sex, partial or total condemnation of the carcass, date of collection, and nature of specimen (*8*). The geographic origins of the cattle could not be evaluated as they were brought to the slaughterhouse by traders from local livestock markets. In the subsample of 199 animals, entire condemnation of the carcass in comparison to partial condemnation occurred more often among Mbororo than Arab cattle (19/75 vs. 11/124, χ^2^, p = 0.002). A higher proportion of Mbororo cattle with bovine TB infection was also observed in Cameroon (*9*); this finding may indicate that Mbororo are more susceptible to M. bovis infection in the 2 Central African countries.

The 201 collected specimens were washed 3 times with sterile, distilled water. Tissue samples were cut into 5 or 6 pieces and put in a sterile plastic bag containing 10 mL sterile saline for homogenization. Samples were homogenized in a blender for 1 min; this process was repeated 3 times. Ten milliliters of the suspension was decontaminated with N-acetyl-L-cysteine sodium hydroxide (0.5% NALC-2% NaOH) (*10*), and 0.25 mL was injected onto 2 Lowenstein-Jensen slants, l containing glycerol (0.75%) and l containing pyruvate (0.6%). In addition, Middlebrook 7H9 media containing oleic acid-albumin-dextrose-catalase and PANTA (polymyxin, amphotericin B, nalidixic acid, trimethoprim, azlocillin) were injected with 0.5 mL of the decontaminated suspension. Injected media were incubated at 37°C (without CO_2_) for 8 weeks. Growth of mycobacteria was confirmed by smear (stained by the Ziehl-Neelsen method) and acid-fast–positive colonies were subcultured. Three biochemical tests (*11*) were used to distinguish between *M. tuberculosis* complex and nontuberculous mycobacteria. Results were confirmed by real-time polymerase chain reaction (*10*).

Overall, *M. bovis* was isolated from more than one fourth of tissue samples and in 42% of all positive cultures. Significantly more *M. bovis* isolates were obtained from Mbororo zebu (30/75) than from Arab zebu (26/124) (p = 0.004). The difference remained significant when the type of condemnation and type of organ were included in a multivariate logistic regression model.

Spoligotyping, as described (*12*), was used as a tool for identifying *M. bovis* within the *M. tuberculosis* complex (lack of spacers 3, 9, 16, and 39–43) but also yielded insights into the epidemiology of *M. bovis*. In total, 12 different spoligotypes were found among the 55 *M. bovis* isolates; 51 (92.7%) of 55 isolates were in 8 clusters (>2 strains), which showed a homogenous population structure (Figure).

**Figure:**
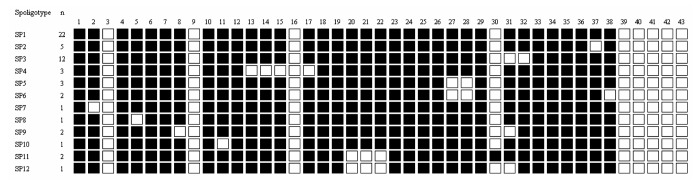
Spoligotypes obtained from 55 *Mycobacterium bovis* isolates from Chadian zebus.

The predominant spoligotype in our study was SP1, with a cluster of 22 strains (40%), as was the case in the study of Cameroon (*7*). SP1 that lacks spacer 30 corresponds to C1; 2 other clusters described in Cameroon (C1 and C5) were also found in Chad (SP2 and SP4). The finding of a high proportion of the same spoligotypes in the 2 countries indicates cross-border movement of cattle. A substantial degree of recent transmission of *M. bovis* strains among cattle is supported by the apparently high prevalence (7%) of TB-like lesions at the slaughterhouse in N'Djaména. However, the homogeneity of bovine strains could also be due to the absence of introduction of new spoligotypes in this particular area. Certain Cameroonian clusters (C7, C8, C9, and C10) (*7*) were only detected in the Adamaoua region, not in northern Cameroon or our Chadian study. The established measures of the Cameroonian government to prevent movement of cattle between the Adamaoua and the 2 northern regions appear effective. As to other neighboring countries, a recent publication describes 15 *M. bovis* isolates from cattle in Nigeria, and these also lack spacer 30 (*13*). This feature seems to be a characteristic of *M. bovis* strains in Central Africa.

Fifteen strains (8 from Arab and 7 from Mbororo zebu) were typed with the IS*6110* restriction fragment length polymorphism (*14*) method, of which 11 and 4 isolates contained 2 or 1 band, respectively (data not shown). Therefore, Chadian *M. bovis* strains belong to low IS*6110* copy number strains. Strains lacking spacer 30 had a band at 1.9 kb, in accordance with the findings in Cameroon (*7*). No association was found between the number of bands and the cattle breed. IS*6110* typing indicated 6 clusters and, thus, was of lower discriminatory power than spoligotyping. In a recent study, variable number of tandem repeat typing was more discriminatory for Chadian *M. bovis* strains than IS*6110* and spoligotyping (*15*).

## Conclusions

The first mycobacterial laboratory established in Chad confirmed bovine TB in Chadian herds by culturing and characterizing *M. bovis*. A high ongoing and cross-border transmission of *M. bovis* in cattle is suspected, but further molecular epidemiology studies are needed to analyze its modes and risk factors. The apparently higher susceptibility of Mbororo zebus to *M. bovis* infection should be followed-up with immunologic assays.
